# P2Y_12_ Inhibition in Murine Myocarditis Results in Reduced Platelet Infiltration and Preserved Ejection Fraction

**DOI:** 10.3390/cells10123414

**Published:** 2021-12-04

**Authors:** Sarah Nasreen Schmidt, Wilfried Reichardt, Beat A. Kaufmann, Carolin Wadle, Dominik von Elverfeldt, Peter Stachon, Ingo Hilgendorf, Dennis Wolf, Timo Heidt, Daniel Duerschmied, Karlheinz Peter, Christoph Bode, Constantin von zur Mühlen, Alexander Maier

**Affiliations:** 1 Heart Center Freiburg University, Department of Cardiology and Angiology I, Faculty of Medicine, University of Freiburg, 79106 Freiburg, Germany; sarah.93@gmx.de (S.N.S.); carolin.wadle@uniklinik-freiburg.de (C.W.); peter.stachon@uniklinik-freiburg.de (P.S.); ingo.hilgendorf@uniklinik-freiburg.de (I.H.); dennis.wolf@uniklinik-freiburg.de (D.W.); timo.heidt@uniklinik-freiburg.de (T.H.); daniel.duerschmied@uniklinik-freiburg.de (D.D.); christoph.bode@uniklinik-freiburg.de (C.B.); constantin.vonzurmuehlen@uniklinik-freiburg.de (C.v.z.M.); 2 University Medical Center Freiburg, Department of Radiology–Medical Physics, Faculty of Medicine, University of Freiburg, 79106 Freiburg, Germany; wilfried.reichardt@uniklinik-freiburg.de (W.R.); dominik.elverfeldt@uniklinik-freiburg.de (D.v.E.); 3 German Consortium for Translational Cancer Research (DKTK), 69120 Heidelberg, Germany; 4 German Cancer Research Center (DKFZ), 69120 Heidelberg, Germany; 5 Department of Cardiology, University Hospital Basel, University of Basel, 4031 Basel, Switzerland; beat.kaufmann@usb.ch; 6 Medical Center Mannheim, Department of Cardiology, Medical Faculty Mannheim, Haemostaseology and Medical Intensive Care University Heidelberg University, 68167 Mannheim, Germany; 7 Baker Heart & Diabetes Institute, Melbourne, VIC 3004, Australia; karlheinz.peter@baker.edu.au

**Keywords:** myocarditis, molecular imaging, activated platelets, echocardiography, P2Y_12_ inhibition

## Abstract

Previous mouse studies have shown the increased presence of platelets in the myocardium during early stages of myocarditis and their selective detection by MRI. Here, we aimed to depict early myocarditis using molecular contrast-enhanced ultrasound of activated platelets, and to evaluate the impact of a P2Y_12_ receptor platelet inhibition. Experimental autoimmune myocarditis was induced in BALB/c mice by subcutaneous injection of porcine cardiac myosin and complete Freund adjuvant (CFA). Activated platelets were targeted with microbubbles (MB) coupled to a single-chain antibody that binds to the “ligand-induced binding sites” of the GPIIb/IIIa-receptor (=LIBS-MB). Alongside myocarditis induction, a group of mice received a daily dose of 100 g prasugrel for 1 month. Mice injected with myosin and CFA had a significantly deteriorated ejection fraction and histological inflammation on day 28 compared to mice only injected with myosin. Platelets infiltrated the myocardium before reduction in ejection fraction could be detected by echocardiography. No selective binding of the LIBS-MB contrast agent could be detected by either ultrasound or histology. Prasugrel therapy preserved ejection fraction and significantly reduced platelet aggregates in the myocardium compared to mice without prasugrel therapy. Therefore, P2Y_12_ inhibition could be a promising early therapeutic target in myocarditis, requiring further investigation.

## 1. Introduction

Myocarditis represents a challenging diagnosis due to the heterogeneity of its clinical manifestations and the lack of sensitive and specific diagnostic tools, including the current gold standard endomyocardial biopsy [1]. Yet it remains a common, at times deadly, entity, being found in 2–42% of autopsies from young adults with sudden cardiac death [2,3] and often leading to lasting functional impairments such as dilated cardiomyopathy with a poor prognosis [4,5]. The underlying etiology is mostly viral [6,7,8], but the disease can, in fact, originate from a variety of infectious and toxic agents, autoimmune disorders, and systemic diseases [9]. Clinical symptoms range from asymptomatic patients to acute heart failure. Elevation of cardiac enzymes, unspecific electrocardiogram (ECG) alterations, or cardiac dysfunction may occur, but may be absent as well [9]. Either way, findings are mostly unspecific and not helpful in discriminatory testing regarding differential diagnosis. Thus, there is a need for new approaches that allow reliable detection of myocarditis in its early course.

A recent study by our group showed that platelets emerge in the myocardium at an early time point, peaking at day 2 of the inflammation in a mouse model of experimental autoimmune myocarditis (EAM), and that myocarditis can be detected by a platelet-specific contrast agent with MRI, which targets the “ligand-induced binding site” (LIBS) of the activated GPIIb/IIIa-receptor. Additionally, platelets were found in human endomyocardial biopsy specimens of myocarditis [10]. As the role of platelets is not limited to thrombotic events but rather involves interaction with endothelial cells [11,12] and the immune system [13,14,15,16,17,18] as well as the initiation of wound healing [19], their pathophysiological function in myocarditis is of fundamental interest.

As it is noninvasive, rather cheap, and easily accessible, targeted molecular contrast-enhanced echocardiography would be a promising tool for early diagnosis of myocarditis, before functional impairment occurs, allowing for specific treatment initiation. Hence, in this study we investigated the relationship of impaired cardiac function and platelet presence in the myocardium in myocarditis, tested a platelet-specific ultrasound contrast agent by targeting the “ligand-induced binding sites” of activated platelets with microbubbles, and evaluated the potential benefit of P2Y_12_ receptor inhibition on cardiac function in a myocarditis mouse model.

## 2. Material and Methods

**Figure 1:**
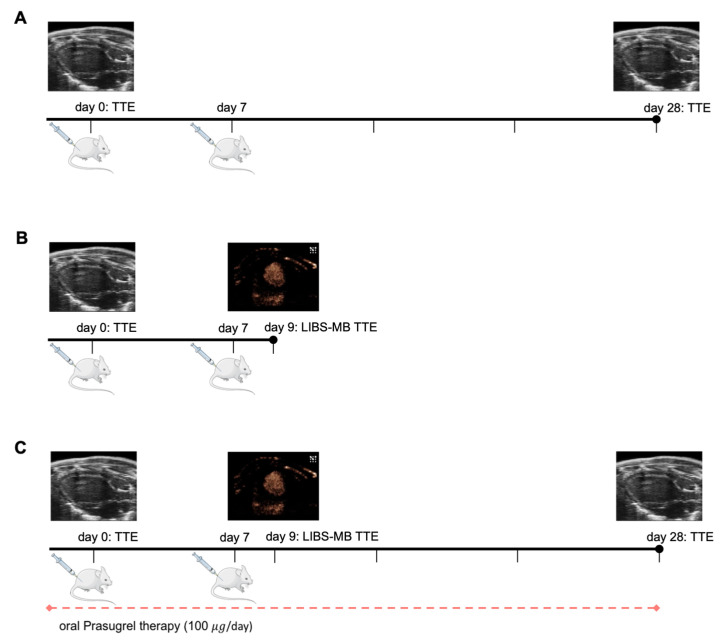
Study design. (**A**) Cardiac function trial: Porcine cardiac myosin was injected subcutaneously (s.c.) in BALB/c mice with CFA (myocarditis group) and without CFA (control group) at day 0 and day 7. Echocardiography to evaluate the left ventricular ejection fraction (EF) was performed on day 0 and day 28. Thereafter, mice were sacrificed for histology. (**B**) LIBS-MB trial: EAM induction (Myocarditis group) at day 0 and day 7 alongside native echocardiography on day 0 and day 9 with contrast echocardiography using LIBS-MB/control-MB on day 9. (**C**) Prasugrel therapy trial: EAM was induced at day 0 and day 7 combined with 1 month of oral prasugrel application by daily oral gavage. Native echocardiography was performed on day 0, day 9, and day 28. Contrast echocardiography using LIBS-MB/control-MB was performed on day 9.

An overview of the study design is given in Figure 1.

### 2.1. Animal Model

All animal experiments were performed in obedience to the German animal protection law, adhering to the standards of good animal practice as defined by the Federation of Laboratory Science Associations (felasa) and the National animal welfare body GV-SOLAS. At the end of the experiments, animals were euthanized by cervical dislocation. Studies were undertaken after the approval of the local authorities and the “Animal Experiment Commission Freiburg” (“Tierversuchskommission Regierungspräsidium Freiburg”) with the animal experiment permission number 35-9185.81/G-17/98.

#### 2.1.1. Myocarditis Induction

Seven-week-old male BALB/c-mice (Charles River Laboratories, Sulzfeld, Germany or Janvier, Le Genest-Saint-isle, France) were subcutaneously injected in the neck with an emulsion of porcine cardiac myosin, 100 µg per injection (Sigma-Aldrich Company, St. Louis, MO, USA, Ref: M0531), dissolved in saline and complete Freund adjuvant (CFA, Sigma-Aldrich Company, St. Louis, MO, USA, Ref: F5881) at day 0 and day 7. Control mice were injected with myosin in saline for sham myocarditis induction.

#### 2.1.2. Prasugrel Therapy

To evaluate potential benefits of P2Y_12_ receptor inhibition in myocarditis, mice underwent myocarditis induction and received oral prasugrel therapy for 29 days. Mice were randomly assigned to prasugrel therapy. A suspension of 10 mg of fine-ground prasugrel (Efient 10 mg, Daiichi Sankyo Europe GmbH, München, Germany) with 5 mL of dimethyl sulfoxide (DMSO, Sigma-Aldrich Company, St. Louis, MO, USA) and 10 mL of tap water was prepared. Then, 150 μL of this suspension (=100 µg prasugrel per dose) were administered by oral gavage (Fine Science Tools GmbH, Heidelberg, Germany, Art. # 18060-20). The prasugrel dose was chosen based on available literature using 3–5 mg/kg body weight for reliable platelet inhibition in mice [20]. On day –1, two loading doses were applied with an interval of at least 4 h in between. From day 0 to day 28, one dose per day was administered.

### 2.2. Contrast Agent Directed against Activated Platelets (LIBS-MB)

A monoclonal single-chain antibody directed against the “Ligand-induced Binding Site” of the thrombocytic GPIIb/IIIa-receptor (anti-LIBS-antibody), which is exposed only in its active conformation, was conjugated to perfluorobutane-filled microbubbles (MB) using a Biotin-Streptavidin-Biotin-Link.

MB had a mean diameter of 2.987 μm. The lipid shell consisted of Distearoylphosphatidylcholine and Distearoylphosphatidylethanolamine-polyethyleneglycol (PEG)-Biotin. Further, a green fluorochrome, Dioctadecyloxacarbocyanine-perchlorat (DiO, excitation maximum 484 nm, emission maximum 501 nm) was added into the shell to enable subsequent detection of the MB by fluorescence microscopy.

Purification of the anti-LIBS-antibody from Escherichia coli cultures has been previously described [21]. Functional testing was performed with Fluorescence-Activated Cell Sorting (BD FACSCanto^TM^ II, BD Biosciences, San Jose, CA, USA) and showed selective binding of the anti-LIBS-antibody to activated human platelets.

For control purposes, a nonfunctional version of this single-chain antibody (=“control antibody”) was used. This modified antibody has an Arginine exchange in the heavy chain’s RXD motive of the complementary determining region 3 [21].

Biotinylation of the anti-LIBS-antibody/control-antibody followed by a 2-(4′-hydroxyazobenzene) benzoic acid (HABA) Assay was performed using a Sulfo-N-Hydroxysulfosuccinimide (NHS)- Light Chain (LC)-Biotinylation Kit (Thermo Fisher Scientific, Waltham, USA, #21435) according to the manufacturer’s instructions.

The MB were washed several times by adding degassed phosphate buffered saline (PBS) and short centrifugation at 1000 revolutions per minute (rpm) to get rid of excess Biotin and lipids. The MB concentration was then counted in a hemocytometer (Neubauer improved, Paul Marienfeld GmbH & Co. KG, Lauda-Königshofen, Germany) and then incubated with Streptavidin (3 µg Streptavidin/10^7^ MB, Sigma-Aldrich Company, St. Louis, MO, USA, Ref: S4762-5MG) for 10 min at room temperature.

After another washing and counting step, incubation with the anti-LIBS-antibody (7.5 µg anti-LIBS-antibody/10^7^ MB) or control-antibody (1.5 µg control-antibody/10^7^ MB) was performed at room temperature for 10 min followed by a last washing and counting step. Conjugation of the anti-LIBS-antibody to the MB was always carried out the same day of in vivo utilization.

MB conjugated to the anti-LIBS-antibody are referred to as “LIBS-MB” and MB conjugated to the control-antibody as “control-MB”.

The contrast agent was infused into the mice via a 30-cm-long tail vein catheter. For an amount of 5 × 10^6^ MB per bolus, the injected volumes varied from 50–75 µL. Before application, the MB had to be shaken manually in a gentle manner to grant homogenous MB distribution. After the injection, sufficient flushing with saline was done to ensure full-dose administration. In order to prevent degradation of the MB, physical stress was avoided and a perfluorobutane headspace was provided at all times.

### 2.3. Echocardiography

All ultrasound experiments were performed on a Vevo^®^ 3100 (FUJIFILM Visualsonics Inc., Amsterdam, The Netherlands) small animal imaging system.

The mice were anaesthetized with isoflurane (induction 4 Vol%, maintenance 1.5 Vol% in oxygen) and placed in a supine position with extremities extended and fixed on a monitoring pad with feedback heat regulation that also registered breathing and ECG cycles. Body temperature was measured with a rectal probe and upheld with the heat regulation feature of the animal monitoring pad. Eye ointment was applied in order to avoid dehydration-driven corneal damage. A small cotton roll was placed under the right hemithorax to mobilize the heart upwards. After shaving the thoracic region, the pad was tilted towards the mouse’s hind right paw and the transducer was then lowered vertically.

#### 2.3.1. Evaluation of Ejection Fraction

Native B-Mode Echocardiography was performed using the transducer MX550D (transmit frequency 40 MHz). Image frames were acquired in the parasternal long axis (PLA) and the parasternal short axis (PSA). Left ventricular ejection fraction was determined using the tool “LV Trace” from Vevo LAB software (version 3.0.0, FUJIFILM VisualSonics Inc., Amsterdam, Netherlands).

Native B-Mode echocardiography was performed on a control group at baseline pre myocarditis induction and on day 28 after sham myocarditis induction, on a myocarditis group at baseline pre myocarditis induction and on day 28 after myocarditis induction, on a myocarditis group at baseline and on day 9 after myocarditis induction, and on a prasugrel therapy group at baseline, day 9, and day 28 after myocarditis induction (Figure 1).

#### 2.3.2. Evaluation of LIBS-MB Binding by Ultrasound

Contrast Echocardiography in the non-linear contrast (NLC) mode was performed using the transducer MX250 (transmit frequency 20 MHz). The appropriate presets for cardiac-targeted MB imaging were selected (cardiac preset, transmit power: 4%, gate: medium, frame rate: 15–20 frames/sec, clip length: 100 frames, burst duration: 1 sec, burst position: 10%, ECG and respiratory gated imaging) and a midpapillary plane in the PSA was chosen.

After bolus injection and observed wash-in of the MB, imaging was paused for 8 min in order to allow removal of unattached MB from the circulation for reduced background signal as well as to facilitate specific enhancement of LIBS-MB in the myocardium while avoiding ultrasound-related MB destruction.

After 8 min, imaging was resumed and a burst-sequence clip was recorded with 10 frames pre-burst, destruction of all MB in the imaging plane via highly energetic ultrasound pulses (=burst), and 90 frames post-burst signal. The acquired clips were analyzed using the Vevo CQ software (FUJIFILM VisualSonics Inc., Amsterdam, Netherlands) that uses algorithms to linearize the non-linear MB signal. The left ventricular myocardium was defined as the region of interest (ROI) and the mean difference of linearized signal intensity before and after the burst, the “differential Targeted Enhancement” (d.T.E.), was calculated. For pre-burst signal, one of the first five acquired frames was selected, as Kaufmann et al. showed no significant imaging-related MB deterioration in low mechanical index (MI) imaging up to this point [22]. For post-burst signal, several frames were averaged to obtain more reliable values.

Contrast echocardiography was performed in a myocarditis group (Figure 1B) and a prasugrel therapy group (Figure 1C) at an early time point of inflammation. Each mouse received both a LIBS-MB bolus and a control-MB bolus in a randomized fashion. To undermine interferences between the two contrast agents, a pause of at least 15 min with constant imaging and several bursts was put in between. After imaging, each mouse of the myocarditis group received another bolus of either LIBS-MB/control-MB for histological assessment with a waiting period of 8 min disregarding the burst sequence in the end.

### 2.4. Histology

For histological assessment, animals were sacrificed; the hearts were removed, gently flushed with saline, and cut into halves. The hearts were then embedded in optimal cutting temperature (O.C.T.) compound, frozen at –20 °C, cut into 7 µm sections, and fixated with Acetone at −20 °C for 10 min.

#### 2.4.1. Inflammation and Necrosis

Hematoxylin-Eosin (HE) staining (25% Gill III-Hematoxylin-solution, Sigma-Aldrich Company, Ref: GHS332-1L and Eosin G 0.5% solution, Carl Roth GmbH, Ref: X883.1) was performed for assessment of inflammatory infiltrates and necrosis.

For each mouse, 12 different heart sections were evaluated in 20× magnification in a non-blinded fashion. The percentage of the affected myocardium was calculated based on the number of quadrants with inflammatory infiltrates.

#### 2.4.2. Platelet Count

CD41 Immunohistochemistry was performed according to established protocols for assessment of the platelet amount in the myocardium [10]. Briefly, sections were incubated with a rat anti-mouse CD41 IgG1 antibody (Gene Tex, Inc., Irvine, CA, USA, Ref: GTX76011) as primary antibody and an isotype control antibody (Bio-Rad Laboratories, Watford, UK, #MCA 1211) as negative control. A biotinylated Rabbit anti-Rat IgG (Vector Laboratories, Burlingame, CA, USA, BA-4001) for secondary staining was used. For detection of the secondary antibody, Vectastain® ABC Kit (Vector Laboratories, Burlingame, CA, USA, Ref: PK-6100) with the substrate VECTOR® Red (Vector Laboratories, Burlingame, USA, Ref: SK-5100) and Levamisole (Dako North America, Inc., Carpinteria, CA, USA, Ref: X3021) was used.

Platelets were counted in two representative pictures of 20× magnification of the inflamed myocardium of two different CD41-stained slices in a non-blinded fashion, as described before [10,23].

#### 2.4.3. Evaluation of LIBS-MB Binding by Histology

For assessment of LIBS-MB binding in the myocardium, sections from the myocarditis group, which had received LIBS-MB/control-MB, were embedded in Roti®-Mount FlourCare fluorescence medium (Carl Roth GmbH, Karlsruhe, Germany). A fluorescence filter set 38 (BP 470/40, FT 495, BP 525/50) by Zeiss, Germany, was used.

For each mouse, 30 fields of view (FOV) in 40× magnification from 12 different heart sections were evaluated in fluorescence microscopy and the average amount of microbubbles/FOV was calculated.

### 2.5. Statistical Analysis

Statistical analysis was supported by Prism (Version 8.0.2, GraphPad, San Diego, CA, USA). All analyses were conducted using a *p* ≤ 0.05 significance level. The graphs are displayed as dot plots with the group mean indicated.

For the comparison of two paired samples with normal distribution, a paired t-test was used. When symmetrically distributed, a Wilcoxon test was used. For the comparison of two unpaired samples with normal distribution plus similar variances, an unpaired t-test was used. When variances differed significantly, Welch’s t-test was used. When there was no normal distribution, a Mann-Whitney test was used.

For multiple comparisons with normal distribution, the Ordinary One-Way ANOVA with Holm–Sidak’s multiple comparisons test/Tukey’s multiple comparisons test as post hoc test was used. For multiple comparisons without normal distribution, a Kruskal-Wallis-test with Dunn’s multiple comparisons test as post-hoc-test was used.

## 3. Results

### 3.1. LIBS-MB Is Not Suitable for Specific Detection of Early Myocarditis by Ultrasound in Mice

**Figure 2:**
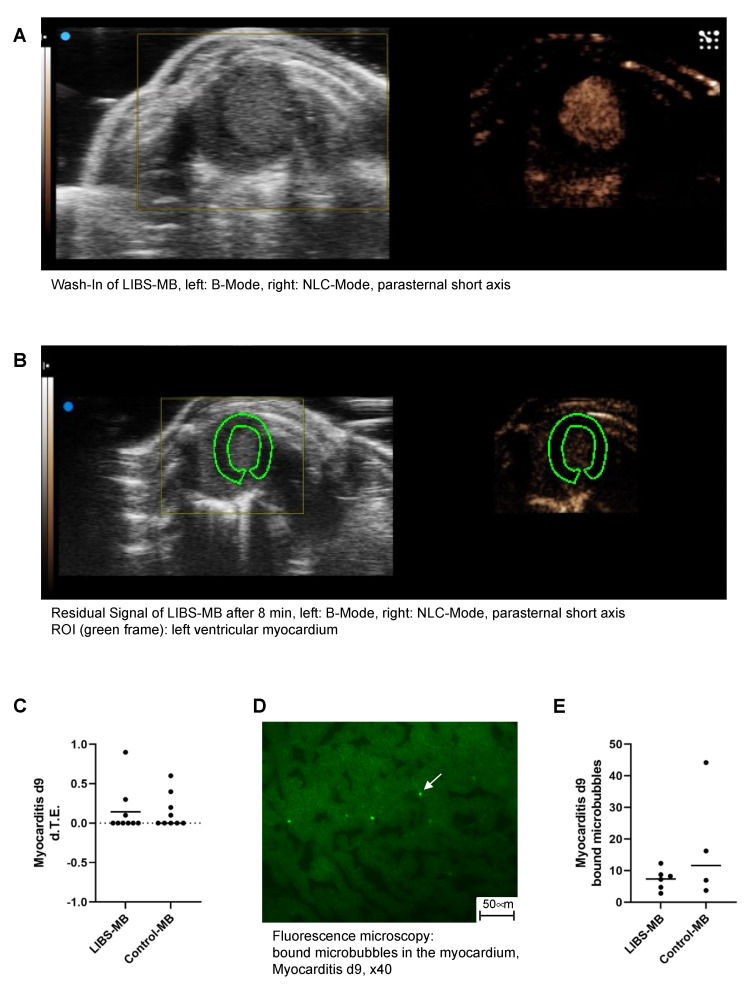
LIBS-MB is not suitable for specific detection of early myocarditis by ultrasound in mice. (**A**) Wash-in of LIBS-MB directly after bolus injection depicted via B-mode on the left and non-linear contrast mode on the right in the parasternal short axis. (**B**) Residual signal of LIBS-MB after 8 min, when imaging was resumed right before bursting depicted via B-mode on the left and non-linear contrast mode on the right in the parasternal short axis. The left ventricular myocardium is shown as ROI within the green frame and shows no macroscopically visible residual signal. (**C**) The differential Targeted Enhancement of both LIBS-MB (*n* = 9) and control-MB (*n* = 9) in the myocardium of myocarditis mice on day 9 obtained by contrast echocardiography shows no selective binding of either contrast agent (Wilcoxon test, two-tailed, paired). (**D**) Fluorescence microscopy in 40× magnification, showing bound microbubbles in the myocardium of myocarditis mice on day 9, as exemplarily shown by the white arrow. (**E**) Counting of bound microbubbles in the myocardium of myocarditis mice on day 9 supports the ultrasound findings: No significant difference between binding of LIBS-MB (*n* = 6) and control-MB (*n* = 4) could be observed (Mann–Whitney test, two-tailed).

Molecular contrast-enhanced echocardiography during the early inflammatory phase of myocarditis did not show selective binding of LIBS-MB in the myocardium (Figure 2A–C). On day 9, mean d.T.E. of LIBS-MB in the myocarditis group was 0.1444 ± 0.3005 arbitrary units (a.u.), whereas the mean d.T.E. of control MB-injected mice was 0.1444 ± 0.2186 a.u., showing no significant difference between the two contrast agents (Figure 2B,C).

Counting fluorescent MB in the myocardium, as seen in Figure 2D, supported these findings as no significant difference in MB binding could be detected between LIBS-MB (7.342 ± 3.248) and control MB-injected mice (17.77 ± 17.93, Figure 2E).

### 3.2. Platelet Infiltration of the Myocardium Occurs before Inflammatory Infiltrates and Reduction of Ejection Fraction in Murine Autoimmune Myocarditis

**Figure 3:**
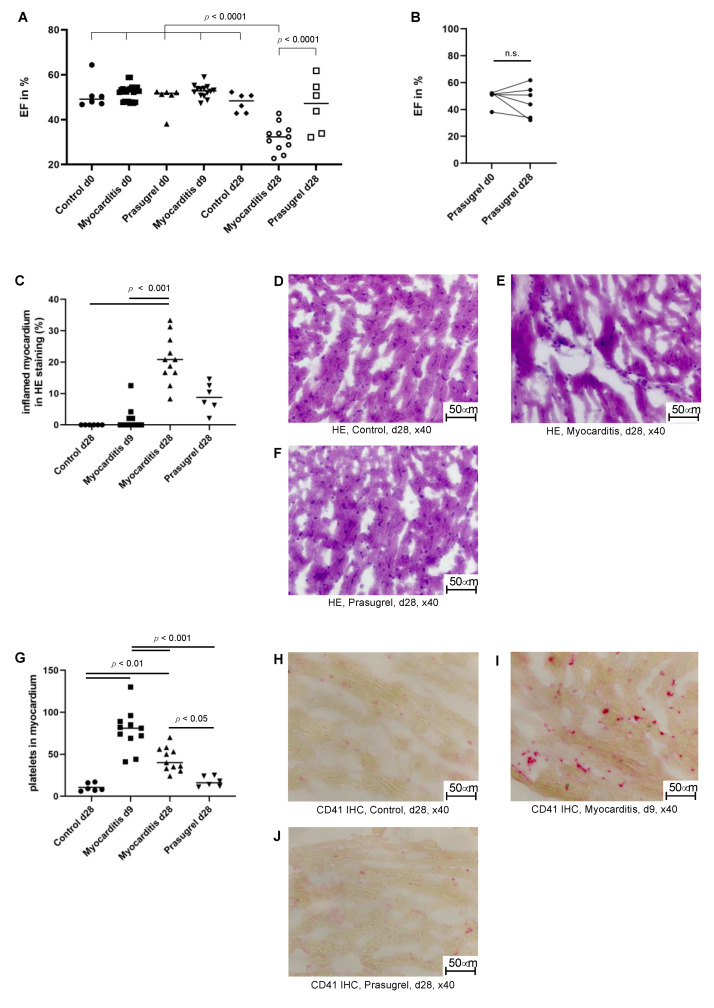
Platelets occur before inflammatory infiltrates and heart failure; P_2_Y12 receptor inhibition with prasugrel limits heart failure and platelet aggregation in the myocardium in murine myocarditis. (**A**) Comparison of the ejection fractions of mice with myocarditis (*n* = 11), mice without myocarditis (*n* = 6), and prasugrel therapy mice (*n* = 6). On day 28, a significantly improved ejection fraction of the prasugrel therapy group was seen (ordinary one-way ANOVA). (**B**) Paired observation of the ejection fractions of myocarditis mice with simultaneous oral prasugrel therapy (*n* = 6) shows a mean ejection fraction on day 28 similar to the baseline level (Wilcoxon test, two-tailed, paired, n.s. (not significant)). At the same time, a widespread distribution is evident with some mice preserving their ejection fraction and some mice deteriorating to the level of myocarditis mice without therapy. (**C**) Inflammatory infiltrates in HE staining tend to be reduced in prasugrel therapy mice (*n* = 6) compared to the myocarditis mice on day 28 (*n* = 11), though the difference is not significant. Myocarditis mice on day 28 exhibit more inflammatory infiltrates than myocarditis mice on day 9 (*n* = 11) and control mice on day 28 (*n* = 6, Kruskal-Wallis test, Dunn’s multiple comparison test). (**D**–**F**) HE staining in 40× magnification showing a representative image of myocardial tissue from control animals, myocarditis mice, and prasugrel therapy mice on day 28 by light microscopy. (**G**) Platelet infiltrates in the myocardium of prasugrel therapy mice on day 28 (*n* = 6) are significantly reduced compared to myocarditis mice both on day 9 (*n* = 11) and day 28 (*n* = 11). The counted amounts of platelets are in the range of control mice (*n* = 6, Ordinary one-way ANOVA, Tukey’s multiple comparisons test). (**H**) CD41 immunohistochemistry in 40× magnification showing few to no CD41-positive aggregates in healthy myocardial tissue from control mice on day 28 by light microscopy. (**I**) CD41 immunohistochemistry in 40× magnification showing a high number of CD41-positive aggregates in myocardium from myocarditis mice on day 9 by light microscopy. (**J**) CD41 immunohistochemistry in 40× magnification showing few to no CD41-positive aggregates in myocardial tissue from prasugrel therapy mice on day 28 by light microscopy.

Mice with late-phase myocarditis (28 days after first injection of myosin and CFA) had a significantly impaired ejection fraction (mean 31.97%; standard deviation (SD) 6.136%) compared to control mice only injected with myosin (mean 47.57%; SD 4.204%) and also compared to mice with early myocarditis (9 days after first injection of myosin and CFA; mean 52.66%; SD 2.874%) (Figure 3A) as ejection fraction on day 9 remained on the baseline level (Figure S1A). However, ejection fraction on day 28 showed a significant relative reduction of 19.7 ± 6.2% as compared to baseline on day 0 (*p* < 0.0001, Figure S1B). Exemplary movie clips of each time point are shown in the Supplemental Material.

In parallel to the decline of the ejection fraction, a significant increase of inflammatory infiltrates and tissue damage can be seen in H&E-stained myocardium at late phases of the myocarditis compared to control and early phases (*p* < 0.001, Figure 3C). Figure 3D shows healthy myocardium of a control mouse. An example of an inflammatory infiltrate in H&E-stained tissue is shown in Figure 2E. Overall, inflammation was only visible to a minor degree in late myocarditis on day 28 as the mean infiltrated myocardium was 20.83 ± 7.569%.

Platelets in the myocardium had already occurred in early phases of the myocarditis before ejection fraction was impaired and infiltrates by H&E staining were observed (Figure 2G). Nine days after induction of myocarditis more platelets were found in the myocardium compared to control animals and animals with late-phase myocarditis 28 days after the induction, as detected by CD41 immunohistochemistry (*p* < 0.0001). Most of the platelet aggregates were located in the interstitial space.

### 3.3. P2Y_12_ Receptor Inhibition by Prasugrel Limits Heart Failure and Platelet Aggregates in the Myocardium in Murine Myocarditis

Mice, which underwent induction of autoimmune myocarditis concomitant to oral prasugrel therapy for 4 weeks, had a preserved mean ejection fraction on day 28 (46.1 ± 11.7%), which did not vary significantly from their baseline values (49.6 ± 5.6%, Figure 3A). The development of ejection fractions within the prasugrel therapy group was widespread, with two mice having a decline similar to the mice in the myocarditis group (EF on day 28: 33.9% and 32.2%), one mouse with moderate deterioration (EF on day 28: 43.8%), and three animals maintaining their ejection fraction in the range of that of control mice with sham myocarditis induction (EF on day 28: 50.7%, 61.8%, and 54.6%, Figure 3B). Overall, prasugrel therapy for 4 weeks led to preservation of the mean ejection fraction (46.1 ± 11.5%) at a similar level to those of control mice with sham myocarditis induction (47.6 ± 4.1%). Mice with myocarditis had a significantly worse ejection fraction compared to myocarditis mice receiving prasugrel therapy on day 28 after induction (*p* < 0.01, Figure 3B).

H&E staining of myocardial sections from prasugrel-treated mice did not show significant differences in infiltrates 28 days after myocarditis induction compared to all other groups (Figure 3C). Examples of H&E-stained myocardial sections and CD41 platelet staining of mice treated with prasugrel are given in Figure 3F,J. The numbers of platelets in the myocardium 28 days after myocarditis induction in prasugrel-treated mice were significantly lower compared to mice not receiving P2Y_12_ receptor inhibition treatment. Platelet burden was comparable to levels in control mice with sham myocarditis induction (Figure 3G).

Platelet-targeted molecular echocardiography with LIBS-MB of prasugrel-treated mice did not differ significantly from mice not receiving prasugrel (Figure S2A). The d.T.E. of LIBS-MB (0.06667 ± 0.08165 a.u.) and control-MB (0.2333 ± 0.3386 a.u.) did also not differ significantly in prasugrel-treated mice (Figure S2B).

## 4. Discussion

This study demonstrates that the peak number of platelets emerges in an early inflammatory phase of murine autoimmune myocarditis, before heart failure by echocardiography and inflammatory infiltrates by H&E staining can be detected. Platelets could not be detected at their peak occurrence time by molecular echocardiography with LIBS-MB. P2Y_12_ receptor platelet inhibition therapy with prasugrel resulted in a significantly preserved ejection fraction and less platelet accumulation.

The time-dependent role of platelets in myocarditis in mice was described before. In the same study, platelet involvement in human myocarditis specimens was shown [10]. The mechanism behind platelet involvement in myocarditis remains speculative. Early P2Y_12_ receptor inhibition with prasugrel resulted in a significantly better mean ejection fraction after 4 weeks of therapy, emphasizing that platelets and the P2Y_12_ receptor may play an important pathophysiological role in the course of the disease. Platelets occur before heart failure is present and may, thus, be part of an early inflammatory cascade in myocarditis.

Platelets interact with the innate and adaptive immune system in multiple ways. Toll-like receptors enable them to recognize pathogen-associated molecular patterns and to react via activation of leucocytes or the thrombotic pathway [13,16]. They interact with the complement system, for instance, by binding the complement factor C3b and inducing the formation of the membrane attack complex [24]. Furthermore, they are able to secrete several inflammatory cytokines [14,15,19] or microbiocidal agents, such as thrombocidin 1 and 2, themselves [17]. Platelets can prepare the endothelium for the recruitment of leukocytes through the expression of soluble and surface-resident CD40 ligand, leading to upregulation of endothelial adhesion molecules as well as endothelial secretion of interleukin (IL)-6, IL-8, and other inflammatory cytokines [11,19,25]. On the other hand, they can directly activate leukocytes by expressing P-selectin on their surface, which leads to binding and rolling of the leukocytes on the platelet-surface [12,19,26]. P2Y12-inhibition suppresses Adenosine diphosphate (ADP)-mediated P-selectin expression [27,28] and CD40L release [29], which results in reduced platelet activation, aggregation, and degranulation. However, unpublished data of our group show a significant reduction of ADP platelet reactivity by aggregometry after prasugrel treatment in C57BL/6 mice.

Coxsackievirus B3 (CVB3)-induced myocarditis leads to formation of platelet-leukocyte aggregates correlating with thrombocytopenia in mice. Platelets do not participate in virus replication but react to CVB3 binding by shifting phosphatidylserine and P-selectin to their surface, enabling platelet–leukocyte interaction. Inhibition of GPIIb/IIIa-mediated platelet activation leads to moderately reduced viral titers [30]. Taking into consideration that a non-infectious autoimmune model of myocarditis was used in our study, the observations with CVB3-induced myocarditis are in line with our results of preserved cardiac function after P2Y_12_ inhibition. However, in the same study, complete platelet depletion resulted in higher viremia and aggravation of myocarditis with an increased mortality, indicating a crucial function of platelets in the antiviral response.

The P2Y_12_ receptor is mainly expressed on platelets, but also on vascular smooth muscle cells and immune cells including mast cells, monocytes, macrophages, and lymphocytes [31]. Inhibiting these immune cells via P2Y_12_ inhibitors may exert anti-inflammatory effects beyond the well-known anti-platelet effects of these inhibitors [32]. Myocardial necrosis was reduced and ejection fraction was preserved in ischemia reperfusion injury in P2Y_12_ knockout mice in a previous study by our group [23]. Whether the beneficial effect on cardiac function is caused by platelet, immune cell inhibition, or a combined effect of both, was not part of this study. Further studies are needed to investigate if the beneficial effect of P2Y_12_ inhibition is limited only to the myocardium or if there are systemic effects, which would affect immunological organs like the spleen and bone marrow. Our results showed a lower platelet immune response in the myocardium after P2Y_12_ receptor inhibition with prasugrel. Shorter prasugrel treatment might be sufficient as well but needs to be tested. Bleeding complications were not observed with prasugrel therapy.

LIBS-MB did not show selective binding compared to control-MB either by ultrasound or histology. This was an unexpected result as the anti-LIBS antibody has already been successfully used for the detection of activated platelets in several studies [10,23,33,34], in particular, for in vivo characterization of ischemia-reperfusion injury after left anterior descending artery (LAD) ligation [23] and detection of arterial thrombosis by MRI [35]. Conjugation to microbubbles does not alter the capacity of the anti-LIBS-antibody to build a solid and specific bond to its epitope in flow chamber experiments: LIBS-MB adhere to both platelet monolayers and microthrombi even under arterial flow conditions. It was even used for in vivo detection of carotid artery thrombosis by ultrasound in mice [34]. Moreover, our group recently used the anti-LIBS antibody coupled to microparticles of iron oxide (LIBS-MPIO) for early detection of autoimmune myocarditis in BALB/c-mice by MRI [10].

This raises the question of why MRI with LIBS-MPIO shows a strong and specific signal effect in myocarditis, whereas ultrasound with LIBS-MB does not. The MB diameter (~5 μm) exceeds MPIO diameter (1 μm) five times. MB usually are not able to leave the intravascular space [36], limiting the range of possible targets to intravascular epitopes. Our hypothesis that inflammation and tissue damage in this mouse model of autoimmune myocarditis produces endothelial leakage large enough for MB to enter the interstitial space to adhere to resident platelets could not be confirmed. Common contrast agents for MRI are capable of diffusion [37].

We chose an established autoimmune model of myocarditis as it shows many pathophysiological similarities to the virus-induced disease but, at the same time, provides biosafety [38,39]. Literature reports that approximately 65% of mice injected with cardiac myosin and CFA develop histological myocarditis [38]. Sampling error and the lack of sensitivity of common histopathological diagnostic tools might be reasons for that [40]. Of note, all of our mice injected with myosin and CFA developed heart failure, as diagnosed by echocardiography. Post-mortem myocardial biopsies from patients with myocarditis have shown a low sensitivity of the Dallas Criteria as the pathological characteristics are focal and do not necessarily involve extensive inflammatory infiltrates [41,42]. Therefore, we compared the mean values of the different groups instead of defining single animals as healthy or diseased by either histology or cardiac function. Our findings of a significantly impaired cardiac function and histological inflammation at late time points in the disease course have been observed in other studies as well [43,44,45]. A recently published study worked with the same animal model as the one used in this study and observed a similar ejection fraction course with maintained good ejection fraction at the early phase and impaired ejection fraction at late phases of murine autoimmune myocarditis [46].

## 5. Limitations

This study has several limitations. Firstly, anesthesia might have a cardio-depressive effect on the ejection fraction [47,48,49]. Literature reports mean ejection fractions of 65% after 5 min of isoflurane anesthesia as compared to 83% in conscious animals [49]. Long-term side effects of isoflurane are not well characterized. However, the anesthesia regimen was as low and short as possible, and all mice underwent the same procedures, with documented similar ejection fractions at baseline for all groups. Secondly, our finding of a beneficial effect of P2Y_12_ receptor inhibition on murine autoimmune myocarditis is based on a small number of animals (*n* = 6). Animals were treated before disease induction. Moreover, only prasugrel as an irreversible antagonist of the P2Y_12_ receptor was tested. Whether reversible antagonists show a similar effect and whether treatment after disease induction is beneficial need to be confirmed. We did not examine the isolated influence of DMSO by means of a vehicle control group. The prasugrel therapy group was directly compared to control mice with sham myocarditis induction. Unpublished data of our group in C57BL/6 mice with myocardial infarction using the same dose of prasugrel dissolved in DMSO like this study did not show influence on the ejection fraction after DMSO-only treatment. Thirdly, effects of microbubbles on inflammation are unknown but they are believed to be safe, with few side effects [50]. Despite ECG- and respiration-gated imaging during the LIBS-MB trial, minor motion artefacts could not be avoided, causing little shiftings of the ROI. Finally, the LIBS antibody concentration to produce the LIBS-MB contrast agent was lower compared to earlier similar ultrasound studies [33,34], but higher than the dosage used in a corresponding MRI studies by our group [10].

## 6. Conclusions

Platelets infiltrate the myocardium in the early course of murine autoimmune myocarditis before detectable heart failure or visible histological inflammation in H&E staining.

Depiction of activated platelets by contrast-enhanced echocardiography using the anti LIBS-antibody coupled to MB in early myocarditis was not successful.

One month of prasugrel therapy in mice with myocarditis resulted in a preserved ejection fraction and less platelet infiltration compared to mice without prasugrel therapy and myocarditis. Further studies need to be conducted to understand the mechanism(s) behind this potentially beneficial role of P2Y_12_ inhibition in myocarditis.

## Data Availability

The data underlying this article are available in the article and in its online supplementary material and will be shared on reasonable request to the corresponding author.

## Supplementary Materials

The following are available online at https://www.mdpi.com/article/10.3390/cells10123414/s1, Figure S1: Cardiac function myocarditis mice; Figure S2: LIBS-MB contrast ultrasound in prasugrel treated mice; Videos S1–S3: Cardiac function of myocarditis, control and myocarditis with prasugrel therapy mice.
