# Supplementary material for: P2Y12 Inhibition in Murine Myocarditis Results in Reduced Platelet Infiltration and Preserved Ejection Fraction
Source: Cells. 2021 Dec 4;10(12):3414. doi: 10.3390/cells10123414 (PMC8699761; doi:10.3390/cells10123414)
Supplement: Supplementary file 1 [file cells-10-03414-s001.zip › cells - 1445727-sup-.pdf]

SUPPLEMENTAL MATERIAL

Supplement Figure S1

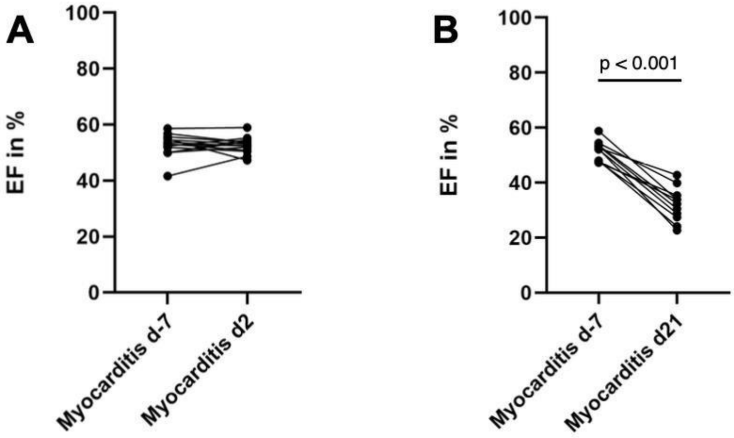

Figure S1: Cardiac function myocarditis mice. **(A)** Paired observation of the development of cardiac function following myocarditis induction reveals a stable ejection fraction at the early phase of myocarditis (day 2) at the level of baseline values (n=14) (t test, two-tailed, paired). **(B)** Paired observation of the development of cardiac function following myocarditis induction reveals a significant deterioration of the ejection fractions at late phases of myocarditis (day 21) compared to baseline values (n=11) (t test, two-tailed, paired).

Supplement figure 2

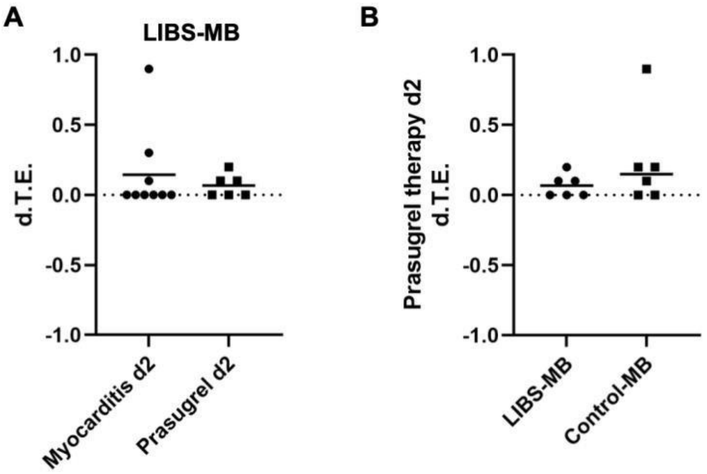

Figure S2: LIBS-MB contrast ultrasound in prasugrel treated mice. **(A)** Myocardial differential Targeted Enhancement of LIBS-MB in contrast enhanced echocardiography on day 2 did not differ significantly between myocarditis mice (n=9) and prasugrel treated myocarditis mice (n=6) (Mann-Whitney test, two-tailed). **(B)** Neither LIBS-MB (n=6) nor control-MB (n=6) showed selective myocardial binding in contrast enhanced echocardiography of prasugrel treated myocarditis mice on day 2. There was no significant difference in the differential Targeted Enhancement of LIBS-MB and Control-MB (Wilcoxon test, two-tailed, paired).

Supplemental video clips: Cardiac function of myocarditis, control and myocarditis with prasugrel therapy mice

(A) An exemplary video showing the cardiac function of a control mouse on day 21 in the parasternal long axis.

(B) An exemplary video showing the cardiac function of a myocarditis mouse on day 21 in the parasternal long axis. A visually impaired ejection fraction and regional wall motion abnormalities are evident.

(C) An exemplary video showing the cardiac function of a prasugrel treated myocarditis mouse on day 21 in the parasternal long axis. Cardiac function is improved compared to myocarditis mice without treatment.
